# Is the Effect of Body Mass Index on Hypertension Modified by the Elevation? A Cross-Sectional Study of Rural Areas in Japan

**DOI:** 10.3390/ijerph14091022

**Published:** 2017-09-07

**Authors:** Tsuyoshi Hamano, Yoshiya Shiotani, Miwako Takeda, Takafumi Abe, Kristina Sundquist, Toru Nabika

**Affiliations:** 1 Department of Sports Sociology and Health Sciences, Faculty of Sociology, Kyoto Sangyo University, Motoyama, Kamigamo, Kita-ku, Kyoto 603-8555, Japan; shiotani@cc.kyoto-su.ac.jp; 2 Department of Functional Pathology, Shimane University School of Medicine, 89-1 Enya-chou, Izumo, Shimane 693-8501, Japan; nabika@med.shimane-u.ac.jp; 3 Center for Community-Based Health Research and Education (CoHRE), Organization for the Promotion of Project Research, Shimane University, 223-8 Enya-chou, Izumo, Shimane 693-8501, Japan; cohre1@med.shimane-u.ac.jp (M.T.); t-abe@med.shimane-u.ac.jp (T.A.); 4 Center for Primary Health Care Research, Lund University, Clinical Research Centre (CRC), Building 28, Floor 11, Jan Waldenströms gata 35, Skåne University Hospital, SE-205 02 Malmö, Sweden; kristina.sundquist@med.lu.se; 5 Departments of Family Medicine and Community Health and of Population Health Science and Policy, Icahn School of Medicine at Mount Sinai, One Gustave L. Levy Place, Box 1077, New York, NY 10029, USA

**Keywords:** hypertension, body mass index, elevation, rural area, cross-sectional study

## Abstract

Obesity is an established independent risk factor for developing hypertension. A recent study showed that the effect of obesity on hypertension varies by the elevation of the residence area. Thus, we hypothesized that the interaction effect of body mass index (BMI) and elevation has a significant association with hypertension. The first aim of this cross-sectional study was to examine whether BMI was associated with hypertension, after adjustment for covariates. The second aim was to examine whether the interaction term between BMI and elevation was associated with hypertension, after adjustment for covariates. Data were collected from a cross-sectional study conducted in a rural area of Japan in 2016. After excluding participants with missing data (*n* = 2), data from 729 participants were analyzed. We found that BMI was significantly associated with hypertension. In addition, the interaction term between BMI and elevation had a significant association with hypertension. The findings of the present study support the recent evidence that high BMI is an independent risk factor for hypertension, but its effect varies by elevation. Thus, context-specific interventions could be an effective approach to prevent hypertension in this area.

## 1. Introduction

Hypertension is a major public health concern, with a worldwide estimated number of cases of more than 1.1 billion [1]. A previous study estimated that there are approximately 43 million hypertensive patients (23 million men and 20 million women) in Japan [2]. Obesity is an established independent risk factor for developing hypertension [3]; a recent meta-analysis reported reductions in systolic blood pressure (SBP) and diastolic blood pressure (DBP) of approximately 1 mm Hg for each kilogram of weight loss [4]. A recent study also focused on a new attention to this issue: The effect of obesity on hypertension may not be uniform [5].

A study conducted in 2016 pointed out that the effect of obesity on hypertension varies by elevation [5]. This study showed unique results in that the population-attributable fraction of obesity in areas with low elevation was 36.3%, but that in areas with high elevation was only 22.3% [5]. The authors concluded that the development of interventions designed to address hypertension could be focused on obesity but should also entail different strategies according to the environmental setting, such as elevation [5]. In recent years, research focused on the environmental setting (e.g., elevation) and lifestyle-related disease has gained momentum, with growing attention being paid to Geographic Information Systems (GIS) [6]. This is because GIS allow us to create variables that determine residential environment (e.g., elevation) on the basis of a participant’s longitude and latitude information [7].

Given this evidence, we hypothesized that the interaction effect of body mass index (BMI) and elevation has a significant association with hypertension. To the best of our knowledge, no previous studies have examined this association in a rural area of Japan. The first aim of this cross-sectional study was to examine whether BMI was associated with hypertension, after adjustment for covariates. The second aim was to examine whether the interaction term between BMI and elevation was associated with hypertension, after adjustment for covariates.

## 2. Materials and Methods

### 2.1. Study Design

Data were collected from a cross-sectional study conducted in 2016. The present study was a part of a Shimane Center for Community-Based Health Research Education (CoHRE) Study that was designed to examine the determinants of lifestyle-related diseases, including hypertension [8,9,10,11,12]. The Shimane CoHRE study was conducted by Shimane University in collaboration with a health examination program that involved the town of Ohnan in Japan. This town is located in a rural area in the southern part of the Shimane prefecture, Japan. Health examination programs are available once a year for residents in this town between 40 and 74 years of age who are covered by National Health Insurance. The residents have two options when they wish to undergo a health examination. They may participate in a group examination conducted at public health centers, or they may receive an individual examination conducted at medical institutions. Shimane CoHRE Study was permitted to use data from a group examination for the analysis. In 2016, participants in the group examination were recruited and 731 of the residents (mean age = 65.7 ± 7.3 years, 294 men and 437 women) participated in this examination. After excluding participants with missing data (2 participants), this study analyzed data from 729 participants.

The Ethics Committee of the Shimane University School of Medicine approved the study protocol in 2016 (number 2227, 5/12). Written informed consent was obtained from all participants.

### 2.2. Hypertension

Hypertension was defined according to the data obtained via face-to-face interviews conducted by trained staff and seated blood pressure (BP) measurements. The participants were asked the following question: “Do you currently take a medicine for hypertension?” In our analyses, we used the following definitions: (1) use of antihypertensive medication (Definition 1) and (2) use of antihypertensive medication or BP ≥ 140/90 mm Hg for SBP/DBP (Definition 2).

### 2.3. Body Mass Index (BMI)

Height and weight were measured in the health examination, and BMI was calculated as the weight divided by the height squared (kg/m^2^). We divided BMI into two categories [3]: <25 and ≥25 (overweight/obese).

### 2.4. Elevation

The Geographic Information Systems (ArcGIS, version 10.0, Environmental Systems Research Institute, Redlands, CA, USA) was used to estimate elevation based on the participants’ addresses. The elevation for each participant was assessed using the ArcGIS ready-to-use dataset of digital elevation models. There is no standard cut-off value to divide high- and low-elevation. Thus, this study used median value (median value = 258.0 m) to define high- and low- elevation areas, i.e., ≤258.0 m and >258.0 m.

### 2.5. Other Measures

Other measures included the following: age (years, analyzed as a continuous variable), sex (male vs. female), current smoker (yes vs. no), current alcohol consumption (yes vs. no), regular physical activity (engaged in regular physical activity = yes vs. not engaged in regular physical activity = no), treatment for disease (medication for diabetes mellitus and hyperlipidemia, yes vs. no), elevation (≤258 m vs. >258 m), and car driving (driver = yes vs. non-driver = no). Car driving was assessed via the following question: “Do you have a valid driving license and regularly drive a car?” (yes = driver, no = non-driver). Given that public transportation networks are often worse in rural areas than in urban areas, it is difficult for elderly non-drivers to access health-promoting goods, services, and resources related to hypertension [13,14,15].

### 2.6. Statistical Analysis

x^2^ and *t*-tests were used to compare the characteristics of the study participants according to the elevation. A multivariable logistic regression model was performed to derive odds ratios (ORs), 95% confidence intervals (95% CIs), and *p*-values. A *p*-value less than 0.05 was considered statistically significant. All statistical analyses were performed using IBM SPSS Statistics 20 (IBM Corporation, Tokyo, Japan).

## 3. Results

The characteristics of the study participants are shown in Table 1. There was a statistically significant difference between the low- and high-elevation groups in terms of medication for diabetes mellitus. On the other hand, there were no statistically significant differences in the other variables analyzed.

Table 2 shows the results of the multivariable logistic regression analysis with hypertension (Definition 1: self-reported) as the dependent variable. In Model 1, BMI was significantly associated with hypertension (OR = 2.66, 95% CI = 1.79–3.93), after adjustment for potential covariates. Age and medication for diabetes mellitus and hyperlipidemia were also significantly associated with hypertension (OR = 1.07, 95% CI = 1.04–1.10, OR = 1.71, 95% CI = 1.04–2.80, and OR = 2.22, 95% CI = 1.54–3.20, respectively). In Model 2, the interaction term (BMI × elevation) was significantly associated with hypertension (OR = 0.37, 95% CI = 0.17–0.80), after adjustment for covariates.

Table 3 shows the results of the multivariable logistic regression analysis with hypertension (Definition 2: defined by self-reported or measuring BP) as the dependent variable. In Model 1, BMI was significantly associated with hypertension (OR = 3.54, 95% CI = 2.34–5.35) after adjustment for potential covariates. Age and medication for hyperlipidemia were also significantly associated with hypertension (OR = 1.08, 95% CI = 1.05–1.11 and OR = 1.61, 95% CI = 1.12–2.32, respectively). In Model 2, the interaction term (BMI × elevation) was significantly associated with hypertension (OR = 0.34, 95% CI = 0.15–0.78), after adjustment for covariates.

## 4. Discussion

As hypothesized, the interaction term between BMI and elevation had a significant association with hypertension using both definitions (Table 2: OR = 0.37, 95% CI = 0.17–0.80 and Table 3: OR = 0.34, 95% CI = 0.15–0.78). These results suggest that the effect of BMI on hypertension varies by elevation. That is, the BMI of participants residing in low-elevation areas had higher ORs of hypertension than those residing in high-elevation areas.

The present results are consistent with those of a previous study, and indicate that individuals residing in low-elevation areas had a higher population-attributable fraction of obesity than those residing in high-elevation areas [5]. A previous study argued that a possible explanation for this result could be that permanent residence at a high-elevation area is associated with a decrease in both SBP and DBP, perhaps secondary to chronic hypoxemia [5], which could be explained by the fact that chronic hypoxia may bring on a relaxing effect on smooth muscles [16]. The range of high-elevation observed in this study, however, was 259–465 m; thus, it is difficult to support this hypothesis. Another explanation could be the differing socioeconomic status between participants from low- and high-elevation areas. In our study setting, low-elevation areas were more urbanized than high-elevation areas. Therefore, further studies should include socioeconomic variables, such as income, educational attainment, and occupation, in the statistical models to explore this possibility.

Hypertension is a major public health concern to address as the population ages [1]. The present study shows that high BMI is an independent risk factor for hypertension, but that its effect varies by elevation. Our findings lend support to actions where health professionals should consider context-specific target interventions (e.g., focusing on physical activity in low-elevation urban settings and promoting healthy eating in high-elevation settings) [5]. Our previous research conducted in a rural area of Japan also showed that individuals residing in high-elevation areas were more likely to have a higher salt intake [8]. To promote context-specific interventions, further research to examine differences in lifestyle factors between people living in low-elevation areas and those living in high-elevation areas is required.

The present study has several strengths. To the best of our knowledge, this cross-sectional study is the first to examine the interaction effect between BMI and the elevation of the residence area on hypertension in a rural area of Japan. In addition, we used GIS to estimate the elevation of the participant’s residence according to the longitude and latitude. Furthermore, hypertension was defined by current medication history and objectively measured BP.

There are also a number of potential limitations in this cross-sectional study. First, it is difficult to confirm a causal relationship between the independent and dependent parameters. Further longitudinal studies are needed to address this limitation. Second, misclassification may have occurred in the self-reported data as a consequence of recall bias. However, we have no reason to believe that this potential bias differed between the residential areas. Third, the data are not equitably representative. Fourth, our data could not evaluate consistency of participants’ addresses and living address. Finally, our results could be partly explained by other unmeasured risk factors for hypertension, such as smoking and alcohol drinking experience (as opposed to current usage only) and socio-economic status.

## 5. Conclusions

The present study shows that high BMI is an independent risk factor for hypertension, but its effect varies by elevation. Context-specific interventions could be effective in preventing hypertension in this area.

## Figures and Tables

**Table 1:** Characteristics of the study participants.

Variables	Low Elevation *n* = 364		High Elevation *n* = 365		*p*-Value
	*n*	% or Mean (SD)	*n*	% or Mean (SD)	
Hypertension (yes)					
Self-reported (taking antihypertensive medication)	126	34.6	118	32.3	0.513
Self-reported or BP measurement (taking antihypertensive medication or BP ≥ 140/90 mm Hg for SBP/DBP)	186	51.1	164	44.9	0.096
Age (year)	364	67.6 (7.1)	365	67.4 (7.5)	0.711
Sex					0.844
Male	145	39.8	148	40.5	
Female	219	60.2	217	59.5	
Current smoker (yes)	32	8.8	38	10.4	0.458
Current alcohol consumption (yes)	180	49.5	193	52.9	0.355
Regular physical activity (yes)	116	31.9	120	32.9	0.771
Treatment for disease (yes)					
Diabetes mellitus	51	14.0	34	9.3	0.048
Hyperlipidemia	103	28.3	94	25.8	0.439
Car driving (yes)	313	86.0	318	87.1	0.654
Body mass index					0.206
Less than 18.5	25	6.9	19	5.2	
18.5 to <25.0	250	68.7	275	75.3	
25.0 to <30.0	74	20.3	62	17.0	
30 or higher	15	4.1	9	2.5	

SD: standard deviation; BP: blood pressure; SBP: systolic blood pressure; DBP: diastolic blood pressure.

**Table 2:** Multivariable logistic regression analysis with hypertension (Definition 1) as the dependent variable.

Variables	Model 1		Model 2	
	OR	95% CI	OR	95% CI
Age (per 1 year)	1.07	1.04–1.10	1.07	1.04–1.10
Sex (female vs. male)	1.25	0.84–1.84	1.22	0.83–1.81
Current smoker (no vs. yes)	0.98	0.53–1.81	0.96	0.52–1.78
Current alcohol consumption (no vs. yes)	1.13	0.79–1.61	1.16	0.81–1.66
Regular physical activity (yes vs. no)	1.31	0.91–1.88	1.31	0.91–1.88
Treatment for disease (no vs. yes)				
Diabetes mellitus	1.71	1.04–2.80	1.67	1.01–2.76
Hyperlipidemia	2.22	1.54–3.20	2.22	1.54–3.21
Car driving (yes vs. no)	1.27	0.77–2.09	1.28	0.77–2.10
BMI (<25 vs. ≥25)	2.66	1.79–3.93	4.24	2.46–7.29
Elevation (≤258 m vs. >258 m)			1.27	0.87–1.86
BMI (<25 vs. ≥25) × Elevation (≤258 m vs. >258 m)			0.37	0.17–0.80

Independent variables were coded as follows: sex (0 = female, 1 = male), current smoker, current alcohol consumption, medication for disease treatment (0 = no, 1 = yes), regular physical activity, car driving (0 = yes, 1 = no), BMI (0 = <25 vs. 1 = ≥25), and elevation (0 = ≤258 m, 1 = >258 m). Note that 0 was the reference category. OR: odds ratio; 95% CI: 95% confidence interval; BMI: body mass index.

**Table 3:** Multivariable logistic regression analysis with hypertension (Definition 2) as the dependent variable.

Variables	Model 1		Model 2	
	OR	95% CI	OR	95% CI
Age (per 1 year)	1.08	1.05–1.11	1.08	1.05–1.11
Sex (female vs. male)	1.42	0.98–2.06	1.39	0.96–2.01
Current smoker (no vs. yes)	1.08	0.60–1.93	1.07	0.59–1.93
Current alcohol consumption (no vs. yes)	1.12	0.80–1.56	1.16	0.82–1.62
Regular physical activity (yes vs. no)	1.09	0.78–1.53	1.09	0.77–1.53
Treatment for disease (no vs. yes)				
Diabetes mellitus	1.29	0.78–2.15	1.24	0.74–2.08
Hyperlipidemia	1.61	1.12–2.32	1.59	1.10–2.29
Car driving (yes vs. no)	1.28	0.78–2.08	1.26	0.77–2.06
BMI (<25 vs. ≥25)	3.54	2.34–5.35	6.02	3.26–11.1
Elevation (≤258 m vs. >258 m)			1.01	0.71–1.43
BMI (<25 vs. ≥25) × Elevation (≤258 m vs. >258 m)			0.34	0.15–0.78

Independent variables were coded as follows: sex (0 = female, 1 = male), current smoker, current alcohol consumption, treatment for disease (0 = no, 1 = yes), regular physical activity, car driving (0 = yes, 1 = no), BMI (0 = <25 vs. 1 = ≥25), and elevation (0 = ≤258 m, 1 = >258 m). Note that 0 was the reference category. OR: odds ratio; 95% CI: 95% confidence interval; BMI: body mass index.
